# Questions That This Monograph Raises

**DOI:** 10.4103/0973-1229.32158

**Published:** 2007

1. What is the ideal combination of increased investment in biomedical research by sponsors with least conflict of interest in researchers?
2. What should interested parties do to refurbish pharma's image?
3. Is a voluntary moratorium on pharma spending practically possible?
4. How to resolve the basic schism between profit driven industry and welfare driven profession of medicine?
5. How can CPGs be salvaged from ulterior influence?
6. Can guidelines on guidelines help?
7. Is cost-effectiveness of CPGs a practical solution?
8. Is it possible to grade therapies as Most, Moderate and Least Cost Effective?
9. How can Disease specific Foundations be salvaged from sponsor's influence?
10. How can Journals ensure scientific integrity of conflicted authors and nullify untoward influence of sponsors?
11. Are reports like the Task Force on individual and institutional conflicts of interest of the AAMC followed more in their breach?
12. Are the revised ICMJE guidelines adequate to stub conflicted research?
13. Editorials make pious announcements. Do they really influence what researchers do?
14. How do we protect the interests of human research subjects?
15. Do Best Practice Guidelines and Good Publication Practices really help?
16. Marketability is the name of the game, not usefulness. How do we ensure the latter, even as the former is forwarded?
17. How can we ensure effective traditional therapies remain in use even as new ones are forwarded?
18. How do we ensure non-pharmacological therapies are also forwarded?
19. How can the legitimate thrust of biological psychiatry be encouraged while also forwarding non-pharmacological approaches in psychiatry?
20. What can be done so pharma just cannot consider questionable means as an attractive alternative?
21. How do we expedite scientific self-correction while causing least harm to patients and research subjects?

